# Ethnicity, gender, and migration status: Applying intersectionality methodology to explore barriers to equitable health systems for maternal and newborn health among immigrant populations in Masindi, Uganda

**DOI:** 10.1097/MD.0000000000029698

**Published:** 2022-07-29

**Authors:** Richard Mangwi Ayiasi, Alice Jean Ochola Mangwi, Ruth Young, Christopher Garimoi Orach, Rosemary Morgan

**Affiliations:** a Makerere University, School of Public Health, College of Health Sciences, Kampala, Uganda; b Muni University, Faculty of Health Sciences, Arua, Uganda; c Johns Hopkins Bloomberg School of Public Health, Baltimore, MD, USA.

**Keywords:** ethnicity, gender, immigrant, maternal, newborn

## Abstract

Globally, 298,000 women die due to pregnancy related causes and half of this occurs in Africa. In Uganda, maternal mortality has marginally reduced from 526 to 336 per 100,000 live births between 2001 and 2016. Health facility delivery is an important factor in improving maternal and neonatal outcomes. However, the concept of using a skilled birth attendant is not popular in Uganda. An earlier intervention to mobilize communities in the Masindi region for maternal and newborn health services discovered that immigrant populations used maternal health services less compared to the indigenous populations. The aim of this qualitative study was therefore to better understand why immigrant populations were using maternal health services less and what the barriers were in order to suggest interventions that can foster equitable access to maternal health services. Five focus group discussions (FGDs) (three among women; 2 with men), 8 in-depth interviews with women, and 7 key informant interviews with health workers were used to better understand the experiences of immigrants with maternal and newborn services. Interviews and FGDs were conducted from July to September 2016. Data were analyzed using content analysis and intersectionality. Results were based on the following thematic areas: perceived discrimination based on ethnicity as a barrier to access, income, education and gender. Immigrant populations perceived they were discriminated against because they could not communicate in the local dialect, they were poor casual laborers, and/or were not well schooled. Matters of pregnancy and childbearing were considered to be matters for women only, while financial and other decisions at the households are a monopoly of men. The silent endurance of labor pains was considered a heroic action. In contrast, care-seeking early during the onset of labor pains attracted ridicule and was considered frivolous. In this context, perceived discrimination, conflicting gender roles, and societal rewards for silent endurance of labor pains intersect to create a unique state of vulnerability, causing a barrier to access to maternal and newborn care among immigrant women. We recommend platforms to demystify harmful cultural norms and training of health workers on respectful treatment based on the 12 steps to safe and respectful mother baby-family care.

## 1. Introduction

Globally, 298,000 women die due to pregnancy related causes each year and half of these occur in Africa.^[1]^ Although global maternal mortality has reduced, in sub Saharan Africa the trends have either stagnated or worsened in the last decade.^[1–3]^ The Millennium Development Goal (MDG) target of reducing the maternal mortality rate (MMR) by three-quarters could not be met by the close of 2015 because of the slow per annum rate of decline in MMR.^[2,4]^ In Uganda, maternal mortality has marginally reduced from 526/100,000 to 336/100,000 livebirths between 2001 and 2016.^[5]^

Studies have shown that health facility delivery is an important factor in improving maternal and newborn health outcomes.^[6–9]^ The presence of a skilled attendant during the entire continuum of care for maternal and newborn care, especially during intrapartum, has the greatest potential of reducing maternal and newborn morbidity and mortality because it has triple return on investment: reduces maternal mortality, reduces newborn mortality, and reduces still births.^[10]^ Despite this, the concept of using a skilled birth attendant is not popular in Uganda.^[11, 12]^ While nearly all pregnant women interact with the health system at least once during their pregnancy, only two-thirds deliver with support of a skilled attendant; within the Masindi region, only 37% of women used a skilled attendant at birth.^[5]^ In 2013, an intervention to mobilize communities in Masindi for maternal and newborn health was introduced and the results showed marked improvement in utilization of maternal health services such as antenatal care and health facility delivery. However, nonindigenous immigrant populations were found to use maternal health services less compared to the indigenous populations and the study was not able to explain why. The aim of this qualitative study was therefore to gain a deeper understanding of why immigrant populations were using maternal health services less than the general population and what the barriers were in order to suggest interventions that can foster equitable access to maternal health services.

**Figure 1.**
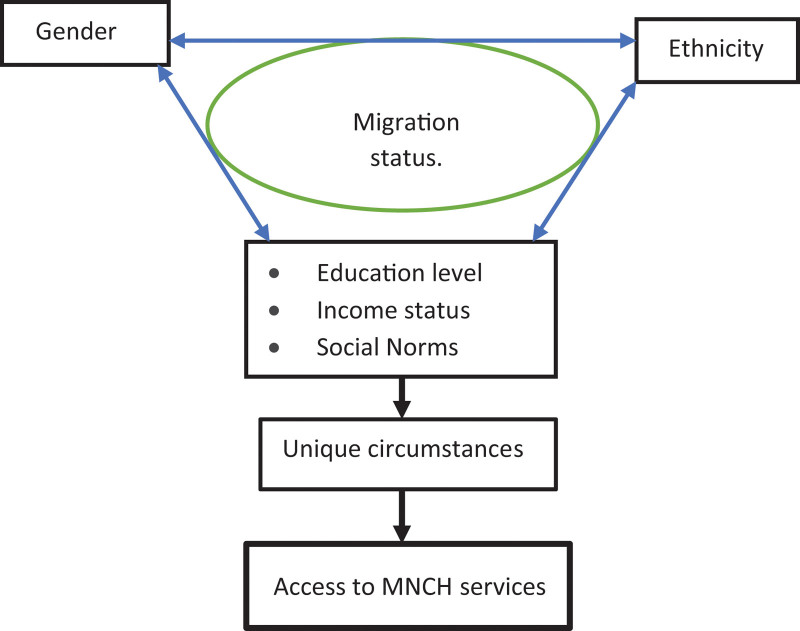
Conceptual framework

Multiple barriers to accessing and utilizing maternal health services are reported in the literature, including poverty, education level, distance from health facility, costs of transport to and from health facility, and cultural and traditional practices.^[13–17]^ However, these barriers are often discussed in relation to the general population. Different groups are likely to have different barriers as a result of multiple factors. We used intersectionality to better understand the specific barriers that immigrant women experienced in accessing and utilizing maternal and neonatal health services. Intersectionality examines how different social stratifiers (such as race, gender, age, immigrant status, etc) intersect with wider systems of power and oppression in society to lead to different experiences of advantage or marginalization and exclusion^[18]^ (Fig. 1: Conceptual framework). Using an intersectionality lens allowed us to examine the experiences of immigrant women during antenatal and delivery care and explore how these experiences interacted with immigrant women’s positionality (gender, migration, and ethnicity) to affect access to maternal and neonatal health services in Masindi district.

## 2. Methods

*Study site*: The study was conducted in *Nyantonzi* parish in Masindi district. This parish was purposively selected because it had the highest density of immigrant population. In Uganda, a parish is a geographical area with a population of approximately 10,000 to 20,000 inhabitants. *Nyantonzi* parish has a population of approximately 15,300 inhabitants, accounting for 4% of the total population of Masindi district. Masindi district consists of 27 parishes, with a total population of 391,900 inhabitants. The local inhabitants of Masindi are predominantly Banyoro, which belong to the large ethnic group known as the Bantu, and they speak the Runyoro dialect. Immigrant populations come mainly from the West Nile districts of Nebbi and Arua, and they belong to the *Sudanic Ma’di* ethnic group. Immigrant populations are essentially economic immigrants seeking job opportunities within the sugarcane plantations of Kinyala Sugar. The *Bantu* and *Sudanic Ma’di* dialects are completely unrelated. In Masindi district, about 35% of women attend 4 antenatal visits and 30% deliver in a health facility, compared to the national average of 57%.^[19]^ Neonatal mortality in the western region of Uganda is 30/1000 live births compared to the national average of 27/1000 live births.^[20]^

*Data collection*: Data were collected through focus group discussions (FGDs), in-depth interviews, and key informant interviews. Five FGDs with immigrant men and women (three with women and 2 with men) were conducted, with each group having 8 participants. Two of the FGDs were conducted with women who had recently delivered and 1 was conducted with pregnant women. The FGDs with men included 1 group whose spouses were pregnant and 1 group whose spouses had recently delivered. All groups were selected based on their *Lugbara* immigrant status. Focus group discussion questions related to perceived barriers to accessing care at the health facility, experiences of care in health facilities (health centers and hospitals), reasons for negative health facility experiences, gender relations at the household level, and suggestions for improving access to and utilization of maternal health care.

In-depth interviews were conducted with lactating women who had experience interacting with the health system during pregnancy or labor. Key informant interviews were conducted with health workers at the referral hospital and 2 health centers in Nyantonzi parish. All interviews lasted approximately 45–60 minutes. Interviews were conducted until saturation was reached.

All interviews were conducted by 2 female social science researchers. One researcher was a gender expert and doctoral student from the school of gender studies at Makerere University, while a second researcher was well versed with the local dialect commonly spoken among the immigrant population. Both researchers were trained on the objectives of the study and were oriented to the question guide.

*Data analysis*: FGDs and in-depth interviews were audiotaped, and translation was performed during interview transcription. Key informant interviews with health workers were conducted and transcribed in English. Thematic analysis was conducted. The first and second authors read the scripts several times and exported them to NVivo software for analysis. Data were coded based on a coding framework which mirrored the themes discussed during the interviews and included intersectional considerations. From the initial codes, texts with similar meaning were grouped together from which subthemes were developed, and subsequently meaningful thematic narrations were developed.

### 2.1. Ethical considerations

This study was approved by the Higher Degrees Research and Ethics Committee of the School of Public Health, HDREC no. 324. Verbal consent to participate in the study was obtained from each study participant. The results of the study were reported to ensure anonymity.

## 3. Results

The results are presented according to the main themes which emerged: we start by looking at how discrimination based on ethnicity was a barrier to access, followed by exploring how ethnicity intersects with other social stratifiers, including income, education, and gender, to compound immigrant women’s marginalization. The details are summarized in Table 1.

**Table 1:** Summary of how ethnicity and gender intersect with other social stratifiers to affect access.

Underlying status	Category	subthemes	Theme (unique circumstance)	Outcome	Mechanism
Being of immigrant status	Ethnicity	Communication barrier	Perceived discrimination & Stigma for being of a particular ethnic group	Low utilization of MCHN care services among immigrant populations settled in sugarcane plantations in Masindi district	First and third delays in accessing care in the three-delay model for access to MNCH care
		Perceived rejection			
		Feeling inferior			
	Gender	Un-supporting male spouses	Late care- seeking practice		
		Silent endurance of labor pains			
	Financial status	Unequal access to finances and decision at household	Low social status		
		Being casual laborers			
		Long working hours in plantations			
	Education status	Low education level			

### 3.1. Ethnicity and immigrant status as a barrier

Ethnic differences were considered an important barrier to accessing maternity care in Masindi. The women perceived deliberate discrimination against them based on their ethnicity. Women explained that health workers identified their ethnicity by looking through their names or their ability to speak the local dialect. Sometimes, a simple greeting in the local dialect is used as a “screening tool” to separate local populations from immigrant populations. Women reported that they were ignored or seen last due to not being able to speak the local dialect:

*“For example, I am a Lugbara, another mother is an Alur, and then a Munyoro mother. If a nurse came and asked us in Runyoro, only one mother would reply* [implying that the Munyoro speaker would reply]. *Those who do not answer back in the language will be abandoned, and the Munyoro mother will be attended to first. At that moment, you will automatically know that she was picked because of tribe, you become timid, they will even start barking at you because you cannot explain your problems, even when nurses talk, you don’t understand”* (**Respondent 1, women FGD 1**)

*“….. A nurse came asked for their books* [meaning medical forms] *which they all handed in, but as she reached her register, she started sorting the books according to the names. Lugbara native names had to be put aside and those of Banyoro aside, she registered those of the Banyoro, took them to the doctors, the doctor started with those names which were presented to him then she registered us later, she even took her time to give the doctor our book, so tribalism is really a disease here it just happened last week”* (**Respondent 6, male FGD 3**).

Women reported feeling helpless due to not being able to predict when a health worker will attend to them, regardless of their medical status:

*“… there you cannot raise a voice…who will listen to you! Even when you are in a critical condition, you have to wait and pray that they may return for you. … Someone will walk away from you especially if they have already worked on the Kiizas and Ayebales* (sarcastically refers to the names used by local ethnic groups)*”* (**Res 2, FGD 1**); *“…on rear chances you can land on Lugbara or Alur nurse* [immigrant ethnicity] *who can rescue you.”* (**Res. 4**, **FGD 1**).

And in some instances, health workers would mock immigrant women, telling them to go and bring their spouses to act as interpreters. These experiences differed from immigrant women who could communicate in the local dialect. According to 1 immigrant woman who could speak the local dialect, ethnic differences create a communication barrier between health workers and women seeking care. She discussed how she can speak the local language and negotiate for appropriate treatment, unlike immigrant women who cannot communicate in the local dialect:

*“But the major problem is language, someone comes to the health centre, cannot speak, Runyoro, Swahili or English. This person only speaks Lugbara, so it becomes difficult to communicate... you know Lugbara and Runyoro have no similarity in any way …when it comes to expressing myself, I will get more drugs than a Lugbara who does not speak the language and cannot express herself, so she is given only what was prescribed yet me I can ask for more”* **(IDI 1**).

As a result of this treatment, some women decided not to seek health facility delivery during labor, as explained by 1 woman:

*“The issue of language has made many mothers abandon the idea of delivering in health centres…in Masindi hospital they will start intimidating you right at the gate … if your name is Ocokoru, Adiru, Candiru* (these are names akin to immigrant populations), *the card will be put aside, even if you are first on the line”* (**Respondent 6, FGD 1 women group**).

### 3.2. Socio-economic status as a barrier

Some immigrant ethnic populations thought they were discriminated against because of their lower socio-economic status and that changing their appearance affected the type of service they received. According to 1 woman, if 1 was well dressed and spoke with a commanding voice, they were likely to be served first compared to the majority of women who were poorly dressed, wore flip-flop sandals, and could not communicate in the local language.

*“In most cases, we do garden work which indirectly affects the way we are treated at the health centre, the way we dress…you may think you have put on your best but when you reach the health centre, you look dirty before the nurses. They say we are not clean, we are not smart, the legs are cracked, we don’t plait our hair …, even the skin texture can tell that you are always in the bush digging, so when they see you, they know you are a farmer, uneducated, they will not get any money from you, so they will not pay any attention to you”* (**Respondent 2, FGD 1 women group**).

Women who appeared to be affluent were better treated by health workers in anticipation of informal payments. Both immigrant ethnic men and women said that the nature of their job, which is considered to be of lower economic status, dictated the way they dressed which ultimately affected the way the health workers treated them.

*“The mode of our work* [plantation workers] *really betrays us, the nurses will look at us, compare and then serve. The ones who are teachers, for example, are served first and quickly so for us we are the last to be catered for”* (R**espondent 4, FGD 3 men group**)

Many of the women migrated to Kinyala for economic reasons and work long hours in sugarcane plantations. Despite this, low income levels for both husbands and wives and high costs of requirements for delivery appeared to be a common reason for the low utilization of delivery services.

“*Our major problem has been money, you may start experiencing labour and you fail to transport yourself to the hospital, even our husbands don’t have proper jobs. This alone has made women to sit back and deliver at home, if there is money every woman will want to deliver in health Centre”* (**Respondent 1, FGD 2 women’s group**).

According to the women, they relied on crops in the gardens for their income, and when it was planting, weeding, or harvest times, they did not have enough money to procure essential requirements for delivery. Moreover, their income was dependent on the harvest season when they were able to sell off some of the harvest.

*“The challenge of money, like right now crops are in the gardens and for us here there is no way one can earn money unless by selling farm produce. At this point if I am pregnant I can’t find money to take me to the health Centre so what one does is to remain strong and deliver at home”* (**Respondent 7, FGD 2, women’s group**)**Education as a barrier**

There were mixed reactions regarding the effect of education status as a barrier to accessing health care among immigrant women. One subgroup of women believed that being of lower education status was the main barrier to access, while another subgroup considered that being an immigrant of lower education status was the main barrier to accessing health care.

*“If you are educated, they know that they can get money from you, so they will attend to you very fast. They even fear educated people because these are informed people who can act against discrimination”* (**Respondent 9, FGD 1 women group**)

*“It is not education status but I think it is because we are from far* [meaning being immigrant populations], *because even uneducated Banyoro are attended to very well”* (**Respondent 1, FGD 1 women group). Gender norms as a barrier**

Many community members perceive it as cowardly for a woman to rush for care at the health facility during childbirth and that endurance of labor pains is a sign of a strong woman.

*“Here the moment you move on to seek care from the health unit during labour, the community members insult you by calling you a coward and saying that you are only married because of the bed service* [meaning sexual intercourse] *but for other issues* [such as enduring labour pains], *you are not a woman”* (**Respondent 5, FGD 4**)

This leads to some women not informing their husbands at the onset of labor; instead they keep quiet until the labor pains intensify which is often within 30 minutes of delivery. For example, 1 woman said she went on normally with her house chores and garden activities for the entire day until the labor pains were strong and she delivered not long after:

*“With my second child I was busy the whole day* [even when the initial labour-pains (latent phase) had already started], *I was busy cleaning my compound, smearing my house and in the afternoon, I went to harvest cassava and, in the evening, labour started* [referring to the strong labour-pains (active phase of labour)] *and by 11 o’clock the baby was out”* (**IDI 2**).

Another gender norm which impacts women’s access to services is pregnancy being seen to be only the purview of women with men having no role to play. When labour sets in most men do not escort their women to deliver at the health center, and some do not provide financial support to allow the women to delivery at a health facility, which can lead to severe complications for women during childbirth.

*“There is a man who comes from Maracha, the wife had wished to give birth at the health centre but the man refused to support her financially. Men think women are naturally strong to deliver at home. Many people approached him to take the wife to the hospital, but he kept dodging after a short while the wife died. … This woman died because the husband could not support her!’* (**Respondent 6, FGD 1 women**).

*“Men here care about the work on the plantation, how much they have earned, it is even worse when the month has ended* [wages are paid at the end of the month]*. What they know is drinking; they do not care to save money for delivery. I saw this when a lady died in this community, being her first child, she did not miss any of her antenatal, she was advised to deliver at the health facility, and had noticed that the child was big. She informed her husband who paid less attention, at the end of it all she died because she was taken late to the hospital not even by the husband but by neighbours”* (**Respondent 2, FGD 1 women**)

## 4. Discussion

There were multiple barriers which affected Immigrant women’s access to maternal and newborn health care within our study. The barriers were influenced by a number of intersecting factors which at times were difficult to disentangle. These included the woman’s ethnicity and immigrant status, socio-economic status, educational level, as well as prevailing gender norms within the community. In the following section, we attempt to explain how ethnicity, immigrant status, socio-economic status, education, and gender intersect to create a complex barrier to accessing maternal and newborn services among immigrant women living in Masindi district. We explain these intersections through the themes of “perceived discrimination” and “gender and cultural norms”.

### 4.1. Perceived discrimination

Discrimination is perceived from different layers of vulnerabilities among immigrant ethnic groups: ethnic differences, low educational levels, and poor socio-economic status. The inability to communicate between immigrant women and predominantly indigenous health workers creates a communication barrier. A systematic review conducted in industrialized Western countries also demonstrated that language proficiency was a deterrent factor in utilizing prenatal care among immigrant women from less industrialized countries.^[21]^ Health workers’ actions to serve first the local populations could be genuinely understood as a means to deal first with people who can be treated quickly and easily, because of the ease of communication, then return to the “more difficult” ones later. However, the downside of this action is that triage is unlikely to be routinely conducted, leading to inadvertent neglect of severe conditions that would otherwise require urgent attention first. A better understanding of the immigrant status of the women could have improved the care offered to them if health workers understood that these were economic immigrants who work to maximize their labor by enduring long working hours for that could partly explain why they arrived to the labor-ward without taking a bathe and usually came straight from the garden.

### 4.2. Gender and cultural norms

Pregnancy and delivery are considered women’s issues. In our study, the male partners disassociated themselves from pregnancy and labor and left the women to seek care on their own. Moreover, as women do not have direct access to household resources, including finance, they often had difficulty paying for services or accessing transportation to get to services.

Societal norms among the immigrant population also dictate that women in labor should endure pain without complaining because suffering in silence during labor is interpreted as a sign of being a strong woman. In contrast, early care-seeking for delivery at a health center is considered to be a sign of cowardice and may attract ridicule from community members on the part of the woman. Early care seeking is also considered to attract unnecessary financial expenditure because of longer hospitalization days. As these immigrant women endured labor pains for a long time and were likely to seek care late, this is likely to necessitate proactive health system interventions—to not only demystify these norms, but also care for women. Proactive health systems should adopt the 12 steps to safe and respectful mother baby-family maternity care released by the international childbirth initiative (ICI). In this framework, steps number 1 and 2 specifically refer to dignified, nondiscriminatory treatment that respects individuals’ customs and values.^[22]^

Using an intersectionality informed approach within this study allowed us to explore how different layers of vulnerabilities intersect to lead to low utilization of maternity services among immigrant women in Masindi district which the previous study could not explain.^[23]^ Intersectionality provides us with a lens to explore the complexity of people’s lived experiences and shows us that barriers to access and utilization of services are not due to 1 or 2 factors alone, but to complex intersecting factors. In turn, this allows us to create more effective services to improve maternal and newborn health services for marginalized groups.

## 5. Limitations

This study is not without limitations. While it was conducted in 1 sub county in which immigrant populations are most concentrated, experiences outside of this country are likely to vary. Due to power dynamics between interviewers and interviewees there is the chance that respondents provided answers that they considered appropriate/correct to the interviewer. In addition, interviews were only conducted with immigrant populations. Obtaining the voices and experiences of nonimmigrants would have helped to provide a comparison.

## 6. Conclusions

This study highlights 2 closely interwoven themes: perceived discrimination along ethnic lines, and gender and cultural norms that relegate maternity issues as women’s-only affairs and reward silent endurance of labor pains. These themes intersect to form a state of vulnerability among female immigrants, creating a barrier to accessing care. Therefore, explaining the delayed care-seeking^[24]^ for maternity services among immigrant women settled around the sugar plantations of Kinyala in the Masindi district.

These barriers to accessing maternity care can be addressed at 2 levels. At the household level, there should be deliberate efforts to engage with men to support their spouses during pregnancy and childbirth, for example, by saving money and preparing for transport to the health facility in case of antenatal care and delivery. At the district political level, local managers together with district health officials should take deliberate efforts to train health workers to be culturally sensitive ^[21]^ to different ethnic groups, especially immigrant populations, to prevent any discriminatory practices. Deliberate training and coaching of health workers on respectful maternity treatment using the patient-centered approach has shown to improve maternity care in Ethiopia ^[25]^ and the intellectual partnership model in Tanzania ^[26]^ whereby facilitators and health workers co-create acceptable approaches to respectful maternity care. Ensuring culturally sensitive respectful maternity care will help to mitigate many of the challenges experienced by participants in this study.

## Author contributions

RMA and AJOM conceptualized the study. RMA conducted the data collection. RMA, AJOM, RY, RM and CGO analyzed the data. RMA, AOM drafted the manuscript. All authors have read and approved the final version of the manuscript.

## Acknowledgments

We acknowledge the contributions of reviews from Professor Sarah Ssalli of Makerere University and Professor Sally Theobald of the Liver School of Tropical Medicine.
